# Concerns over functional experiments, interpretation, and required controls. Reply.

**DOI:** 10.1172/JCI158155

**Published:** 2022-03-01

**Authors:** Wan-Chen Hsieh, Shih-Yu Chen

**Affiliations:** 1 Institute of Biomedical Sciences, Academia Sinica, Taipei, Taiwan.; 2 Genome and Systems Biology Degree Program, National Taiwan University, Taipei, Taiwan.

**Keywords:** COVID-19, Immunology, Innate immunity

## The author’s reply:

We thank Dr. Stanton, Dr. Fielding, and Dr. Wang for their careful reading of our manuscript, especially for their comments on the alterations of the cell surface by SARS-CoV-2 and how these alterations affect NK-related immunosurveillence (1). We agree that a comprehensive profiling of the plasma membrane proteome is essential and that a study similar to that described previously is warranted (2). However, our strategy was to begin by identifying the unique features of NK immunophenotypes that control SARS-CoV-2 and then focus on the relevant ligands. We believe these two directions are complementary and provide different perspectives of how NK cells interact with virus-infected cells.

**Figure 1:**
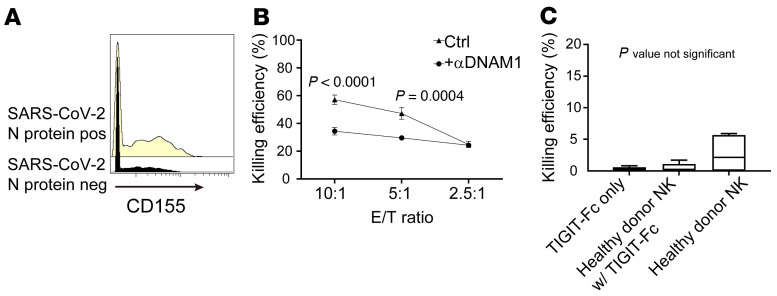
Additional evidence to support the involvement of DNAM1-CD155 interactions in the clearance of SARS-CoV-2. (**A**) Representative histogram of CD155 intensity in SARS-CoV-2 N protein–positive and –negative cells. (**B**) Efficiencies of killing of pseudovirus-infected A549 cells (target [T]) by NK cells (effector [E]) from a healthy donor at 3 effector/target (E/T) ratios with or without anti-DNAM1 antibody. (**C**) Killing efficiency of mock-infected Calu-1 cells by primary NK cells with or without TIGIT-Fc.

Regarding the upregulation of CD155 by SARS-CoV-2, we were also surprised to observe the upregulation of CD155 by pseudovirus that expressed only the spike protein. However, recent reports demonstrated that the spike protein alone or binding of the spike protein to the receptors can trigger signaling in host cells, which is in agreement with our findings that the spike protein is a stressor even in the absence of other viral components (3–6). That downregulation of CD155 is observed upon infection with other viruses (e.g., HCMV) resulting in evasion of NK cell surveillance is not mutually exclusive with our findings. Instead, it further strengthens the importance of investigating NK cell receptor repertoires in detail at the single-cell level. Since NK cells coexpressing DNAM1 and TIGIT, but not DNAM1 alone, are the subsets correlated with the viral clearance, this implies an inhibitory role of CD155 in the context of SARS-CoV-2 infection. We also agree that the differential expression of CD155 that we observed was the cumulative effect of both virus infection and IFN and other cytokines produced following infection. More comprehensive time-course experiments will be required to better clarify these effects, but are beyond the scope of this study. The histogram of absolute levels (mass cytometry) of CD155 is shown in Figure 1A for reference.

To further demonstrate the involvement of DNAM1, we indeed observed a decrease in NK cell killing after incubation with anti-DNAM1 antibody (Figure 1B). The mock-infected controls for the TIGIT-Fc NK experiments are shown in Figure 1C. There were no significant effects of TIGIT-Fc on NK cell killing in mock-infected controls, which demonstrates that the enhancement of NK cell killing efficiency was specific to SARS-CoV-2 virus–infected target cells.

## Version 1 03/01/2022

Electronic publication
